# Supplementary material for: Are carbon emissions trading and green financial instruments synergistic? -Comprehensive quantitative research based on content analysis
Source: PLoS One. 2024 Mar 7;19(3):e0298601. doi: 10.1371/journal.pone.0298601 (PMC10919601; doi:10.1371/journal.pone.0298601)
Supplement: S1 Data — (ZIP) [file pone.0298601.s001.zip › Data/地级市Z_F工作报告.zip!/║╙─╧/░▓╤⌠╩╨/└┤╫╘╣1⁄2╓┌║┼í╛┬φ┐╦╩2╛▌═°í┐_2017.docx]

各位代表：

    现在，我代表市人民政府向大会报告工作，请予审议，并请各位政协委员和其他列席人士提出意见。

    一、2016年工作回顾

    2016年，面对复杂的外部环境和繁重的发展任务，面对“7·19”特大暴雨洪灾，全市人民在省委、省政府和市委的正确领导下，深入贯彻习近平总书记系列重要讲话精神，主动适应经济发展新常态，坚持稳中求进工作总基调，坚持新发展理念，扎实推进“六大工程”“三大攻坚战”，全市经济社会保持平稳健康发展，实现“十三五”良好开局。初步统计，全市生产总值1805.7亿元，增长8%；一般公共预算收入107.3亿元，增长8.7%；规模以上工业增加值765.7亿元，增长7.3%；固定资产投资1901.1亿元，增长13%；社会消费品零售总额662.6亿元，增长11.9%；居民人均可支配收入20513元，增长7.4%。

    （一）稳增长保态势。制定实施稳增长保态势24条和8个专项行动，抓住扩需求、稳企业、防风险等关键环节，促进经济运行总体平稳、稳中向好。着力扩大需求。狠抓重大项目建设，激活民间投资。安阳机场正式获批。国旅安阳城奥特莱斯、永通球墨铸管等300个项目开工。光远电子布、联达数控精密锻造等48个项目竣工或投产。西北绕城高速公路、新东北外环等项目进展顺利。引岳入安一期、万金渠城区段清淤完工，引红入洹工程建成投用。农网改造升级完成投资15.2亿元。688个“461项目”完成投资1073亿元。191个省、市重点项目完成投资549.7亿元。民间投资突破1500亿元。非公有制工业占规模以上工业的比重达到80%。落实“十大扩消费行动”，健康养老、信息服务、旅游等产业快速发展。网络零售额增长297.3%。进出口总值9.5亿美元。接待海内外游客4025万人次，增长17.4%。实施农民进城购房补贴，促进住房消费，市区商品房库存同比下降14.2%。优化企业服务。制定实施企业降本增效28条、金融服务实体经济22条等政策措施，全年新增贷款151亿元，直接融资101亿元。新增各类股权交易市场挂牌企业65家。工银中建、厚德新能源、鑫融基新材料等9支基金组建完成，总规模518亿元。开展政策落实进万企、稳工业“四项对接”、社会保险费“减缓补”等活动，有效帮助企业增效益、稳发展、渡难关。防范化解风险。开展处置非法集资攻坚年活动，贞元、超越案件处置工作取得实质进展。成立安阳中小企业担保公司。累计为中小企业发放应急周转金7.6亿元。在全省首创债权人委员会制度和金融机构共进退机制，防范企业经营风险。

    （二）调结构促转型。深化供给侧结构性改革，扩大有效供给，培育创新动力，促进发展质量效益提升。推动制造业转型提质。制定中国制造2025安阳行动计划。强基精密制造模范工厂、全丰无人机等100个转型升级项目有序建设。安钢冷轧连退镀锌、翔宇康复设备研发及产业化等项目建成投产。工业结构持续优化。淘汰煤炭落后产能45万吨。高成长性制造业增加值增长15.9%。高新技术产业增加值增长17.4%，占规模以上工业增加值的28.1%。产业集聚区规模以上工业总产值增长12.3%。加快服务业优化升级。我市成为首批中国研学旅游目的地城市。红旗渠·太行大峡谷晋升5A级景区。内黄果蔬城、万庄物流园等项目加快推进。保税物流仓库建成投用。新引进金融机构10家。服务业增加值增长10.5%，服务业占生产总值比重42.2%，提高2.7个百分点，对经济增长的贡献率在50%以上。促进新经济加速成长。“互联网+”39个专项行动稳步实施。大数据云计算中心建成，工业云、政务云、民生云全部上线运行。中部易商谷、豫北跨境电商产业园和全省首家“中国生产力众创空间”建成投用。1189个行政村开通电商服务站。推进农业稳中调优。粮食总产219.5万吨。夏粮收购逆市增长10%。新建高标准粮田30.1万亩。农业产业化集群收入282.3亿元。新建、扩建现代农业园区32个，新增面积5.2万亩。创建国家蔬菜标准园13个、养殖示范场18个。成功创建国家农业科技园区。“一环四线两组团”都市生态农业旅游线路基本形成。农村土地规模经营比重达到45%。农产品质量安全监管体系全面加强。实施创新驱动发展。制定加快提升区域创新能力若干意见等8个文件，推动大众创业、万众创新。开展百家院校科技成果走安阳等活动。完成重大科技专项4个。新增院士工作站1家、国家级高新技术企业15家、省级创新研发平台5家。国家质检中心暨安阳综合检测基地主体竣工。岷山公司炼铅新技术及产业化应用项目获得国家科技进步二等奖。

    （三）抓改革促开放。坚持改革开放双轮驱动，拓展发展空间，释放发展活力。深化重点领域改革。调整安阳县、殷都区、北关区、龙安区、文峰区区域管理范围和管理体制，优化发展布局。151项经济体制和生态文明体制改革任务完成98项。政府系统权责清单实现全覆盖，清理行政审批中介服务37项。深化商事制度改革，新登记各类市场主体3.78万户，注册资本增长161%。“营改增”平稳有序，税收征管体制、社会保险费征管体制改革顺利推进。相州农商行挂牌运行，内黄农信社达到农商行组建标准。农村土地承包经营权确权颁证、党政机关公务用车、不动产统一登记、出租车行业等改革基本完成。文旅投资集团投入运营。行政执法、县域医疗综合改革持续深化。落实县以下机关公务员职务职级并行制度和乡镇工作人员补贴。机关事业单位养老保险、科技、教育、文化、供销等改革取得新进展。提升开放招商实效。成功举办安阳融入京津冀协同发展推介会、第八届航空运动文化旅游节，组织参加第十届中国（河南）投洽会等系列活动。北汽镁合金汽车轮毂、嘉士利北方综合生产基地、广州童装产业园等299个新引进项目开工建设。与中冶科工、中车股份、中国建筑等企业合作持续深化。全年引进省外资金563.7亿元，增长8.3%；实际利用外资4.9亿美元，增长5.7%。

    （四）抓创建促协调。强化城市带动，促进城乡统筹。常住人口城镇化率53.8%，提高1.5个百分点。成功创建省级文明城市。完善城市规划。土地利用总体规划调整完善、永久基本农田划定基本完成。中心城区控制性详细规划实现全覆盖。历史文化名城保护、洪河保护与利用、综合管廊等33个专项规划编制完成。安阳古城保护整治复兴规划、洹河景观规划设计、总体城市设计取得阶段性成果。加强城市建设。第八水厂建成投用，市民喝上了丹江水。东风路、解放大道等“八纵六横”道路提升工程竣工。光明路、兴业路等26条道路建成通车。80组智能候车亭、26个公交港湾投用。新增节能环保公交车62台。新增城市公共自行车3500辆。新增供热面积418万平方米。成功入围全省海绵城市试点。提升城市管理。门头牌匾整治全部完成。拆除违法建筑38.4万平方米。规范摊群点、治理小广告、拆除户外广告成效明显。数字化城管二期全面完成，实现建成区全覆盖。统筹城乡发展。渔洋村等13个传统村落改造提升有序推进。美丽乡村建设任务全面完成。新建改建农村公路435公里。改善农村人居环境工作走在全省前列。推进生态建设。营造林19.8万亩。南水北调生态带基本形成。汤河、淇淅河、漳河峡谷3个国家湿地公园建设试点成效初显。光明路南段等42个园林绿化项目完工。新增绿地面积370万平方米。

    （五）强攻坚破难点。深入实施大气污染防治、精准扶贫、国企改革攻坚战，破解发展瓶颈，弥补发展短板。铁腕治理污染。建立 “1+6+7”政策体系，建立五级环保网格，严格落实“六控”措施。依法管控1364家涉气企业。拆改燃煤锅炉62台。305台工业窑炉、62台砖瓦窑炉、28家水泥粉磨站完成提标治理。取缔“小散乱污”企业406家、黑加油站点209个。清除“污染围城”问题3093处。8.5万户城区居民实现以电代煤、以气代煤和洁净型煤替代。全面供应国五标准汽柴油。淘汰黄标车7155辆、老旧车8648辆。夏收、秋收期间秸秆禁烧成效显著。全年空气质量优良天数180天，同比增加14天，PM2.5年日均浓度同比下降5.5%。预计单位生产总值能耗下降5%以上。“两库一泉” 水产养殖得到彻底清理。水资源管理“三条红线”目标全面完成。推进脱贫攻坚。制定出台“五办法、五方案、五专项”等政策措施，实施脱贫攻坚“十大工程”，全面开展“六改一增送温暖”活动。贫困人口转移就业11754人。光伏、特色种养、乡村旅游等产业扶贫覆盖面在80%以上。27324人实现脱贫，87个贫困村脱贫退出，5500人易地搬迁任务顺利完成。加快国企改革。45家国有企业建立法人治理结构或完成公司制改造。豫北棉纺厂等4家改制企业逐一确定遗留问题化解方案。省属国有企业剥离办社会职能走在全省前列。

    （六）惠民生增福祉。扎实办好民生实事，财政民生支出185.5亿元，占总支出的77%。扩大社会就业。城镇新增就业8.29万人。发放创业担保贷款7.93亿元。8200名农民工返乡创业，带动就业3.4万人。农村劳动力转移就业136.8万人。加强社会保障。企业退休人员基本养老金12连增，人均2208元。实施“助保贷”政策。基本医疗保险实现省内异地就医即时结算。城乡居民医保、新农合筹资标准分别提高到每人每年550元和540元。城乡低保月人均补助水平分别提高到240元和132元。社区干部生活补贴每月提高250元。实施困难残疾人生活补贴和重度残疾人护理补贴制度。新开工棚改房9716套，建成5122套。发展公共事业。建设类“全面改薄”项目竣工90个。新建改扩建城镇中小学9所，新增学位1.1万个。第二期学前教育三年行动计划圆满完成。职业教育、民办教育加快发展。卫计融合 “多证合一” 实现全覆盖。搭建“双固定专家义诊平台”。市人民医院搬迁顺利推进。市妇幼保健院儿科综合楼、市第三人民医院门诊综合楼投入使用。“全面两孩”政策平稳实施。推进文化惠民工程，公共文化设施基本实现全覆盖。文物保护工作得到加强。全民健身运动蓬勃开展。妇女儿童、红十字、社会福利、慈善等事业健康发展。老龄、史志、档案和关心下一代工作取得新成绩。维护社会稳定。创新社区管理和服务，创建示范社区60个。深化安全生产专项整治。全面加强食品药品安全监管。依法做好信访稳定工作，集中化解信访积案，维护群众合法权益。强化社会治安综合治理，社会大局和谐稳定。

    过去一年，我们着力加强政府自身建设，深入开展“两学一做”学习教育，持续整治懒政怠政为官不为，强化行政监察和审计监督，专项治理发生在群众身边的不正之风和腐败问题。自觉接受人大法律监督、政协民主监督和社会监督，认真办理人大代表建议和政协提案，支持民主党派、工商联和无党派人士参政议政。坚持依法行政，深化政务公开，政府立法开局良好，法治政府建设取得新进展。军民融合深度发展，军政军民团结更加巩固，第六次荣获全国“双拥”模范城称号。老区建设不断加强。统计、气象、地震、邮政、发展研究、外事侨务、民族宗教等工作取得新成绩。

    各位代表！去年我市发生了历史罕见的“7·19”特大暴雨洪涝灾害。全市上下按照国家防总和省委、省政府重要指示，提前发布预警，紧急动员部署，果断采取措施，及时转移受灾群众，科学实施水文调度，全力组织抢险救灾，88. 86万受灾群众生产生活得到保障，灾后重建有序推进。在这场特大灾害的考验中，安阳儿女众志成城，连续奋战，涌现出无数可歌可泣的英雄模范，形成了团结一心、拼在一线、敢于担当、无私奉献的宝贵精神。

    过去一年取得的成绩，是市委正确领导的结果，是市人大、市政协大力支持的结果，是全市人民团结拼搏的结果。我代表市人民政府，向广大工人、农民、知识分子，向驻安解放军指战员、武警官兵、公安民警及社会各界人士，向关心支持安阳发展的港澳台同胞、海外侨胞和国际友人，表示衷心的感谢！

    各位代表！对照安阳市第十三届人民代表大会第三次会议确定的目标任务，全市固定资产投资、进出口总值、粮食产量、部分环保约束性指标没能完成目标，保障性安居工程配套建设、永明路北段、文昌大道征地拆迁、职工文化体育中心、妇女儿童活动中心等没有完成序时进度。这其中有自然灾害、环保治理、需求不足等因素影响，也有主动作为不够的原因。在此，向各位代表及全市人民表示歉意！

    我们清醒认识到，全市发展中还存在不少困难和问题。一是结构矛盾仍然突出。产业层次低，工业结构重，冶金建材、煤化工等传统产业占比大，产品附加值低，市场竞争力弱。种养业结构不优。现代服务业发育不足。发展质量效益亟待提升。二是动能转换相对滞后。重点行业生产经营困难。科技创新能力不强，创新平台少、人才少，创新型企业规模小、带动力小。新业态新模式尚未成为经济增长的有力支撑。民间投资增长动力不足。个别地方和企业落实新发展理念不够自觉，存在惯性思维和路径依赖，改革相对迟缓。三是环境制约持续加剧。严重雾霾天气频发。钢铁、煤化工、玻璃、铸造、铁合金、水泥、有色金属等高耗能高污染高排放行业占比大，能源结构不合理，涉气涉水企业数量多，污染物排放总量大。环境容不下、资源撑不住、发展保不了的问题突出。四是风险隐患积聚增多。一些企业生产经营成本高、负债率高、亏损严重，经营风险加大。贷款互保链趋紧，潜在区域性金融风险。个别地方政府性投资公司融资结构不尽合理，潜在地方债务风险。社会稳定潜在风险增多。五是发展短板亟待补齐。城乡区域发展不协调。教育、医疗、文化等公共服务体系和道路、公园等公共配套设施建设滞后。就业、社会保障等民生改善任务繁重。脱贫攻坚任务艰巨。六是治理能力仍需加强。一些公务人员依法履职能力不强，推诿扯皮，懒政怠政，不会为、不想为、不敢为甚至乱作为现象依然存在。一些领域不正之风和腐败问题尚未根除。对此，我们一定直面挑战，敢于担当，全力以赴做好政府工作，决不辜负人民的重托！

    二、2017年工作安排

    今年将召开党的十九大，是贯彻市第十一次党代会精神、实施“十三五”规划的重要一年。当前，外部环境依然复杂严峻，国内经济下行压力较大，区域竞争日趋激烈，特别是全市结构调整的阵痛更强、环保倒逼的压力更大、弥补短板的任务更紧。但我市经济运行总体向好的态势没有改变，继续保持平稳健康发展有基础、有潜力、有机遇。供给侧结构性改革深入推进，新技术新业态新模式不断涌现，为我们产业发展和结构调整提供了更多市场机会；河南五大国家战略深入实施，中原城市群发展规划确立了安阳省际区域性中心城市定位，我市成为全省唯一纳入京津冀周边协同发展区的城市，列入首批国家通用航空产业综合示范区，为我们提供了难得政策机遇；全市扩大有效需求潜力很大，尤其是这两年一大批项目启动实施，交通、水利、能源、信息、生态等支撑保障能力明显增强，经济持续发展基础更加坚实。只要我们做好应对复杂严峻局面的充分准备，保持定力，增强自信，奋力拼搏，一定能牢牢掌握工作主动权，开辟发展新境界！

    今年政府工作的总体要求是：全面贯彻党的十八大和十八届三中、四中、五中、六中全会精神，更加紧密地团结在以习近平同志为核心的党中央周围，深入贯彻习近平总书记系列重要讲话精神，深入贯彻党中央治国理政新理念新思想新战略，统筹推进“五位一体”总体布局和协调推进“四个全面”战略布局，认真落实中央和省委经济工作会议、市第十一次党代会各项部署，适应把握引领经济发展新常态，坚持稳中求进工作总基调，突出以新发展理念为引领，突出以提高发展质量和效益为中心，突出以推进供给侧结构性改革为主线，加快实施开放带动、创新驱动、人才强市战略，转型发展，务实重干，全面做好稳增长、促改革、调结构、惠民生、防风险各项工作，保持经济平稳健康发展和社会和谐稳定，迈出“一个重返、六个重大”、建设区域性中心强市坚实步伐，以优异成绩迎接党的十九大胜利召开。

    今年发展的主要预期目标是：生产总值增长8%左右，一般公共预算收入增长8%，全社会固定资产投资增长13%左右，社会消费品零售总额增长11%左右，进出口总值保持稳定，城镇新增就业6.5万人以上，常住人口城镇化率提高1.6个百分点左右，居民收入与经济增长基本同步，居民消费价格涨幅3%左右，节能环保指标完成省定目标，发展质量和效益明显提高，结构性改革取得实质进展。

    做好今年政府工作，要做到“四个统一”：一是坚持稳增长与调结构相统一。从我市看，稳增长是基础，稳得住就能为调结构赢得时间空间，减轻改革“阵痛”；调结构是根本，调得好就能为稳增长创造条件，缩短转型“长痛”。要把握稳中求进工作总基调，以新发展理念为引领，以提高发展质量和效益为中心，打好产业结构优化升级、创新驱动发展、基础能力建设、新型城镇化“四张牌”，把“一个重返、六个重大”目标落在实处。二是坚持扩需求与促转型相统一。发挥投资的关键作用，培育发展新动能。抓住推进供给侧结构性改革主线，落实“去降补”重点任务，打好农村脱贫、国企改革、环境治理、转型发展 “四大攻坚战”，在结构转型、动能转换和方式转变上取得实质性成效。三是坚持抓改革与防风险相统一。扎实推进重点领域和关键环节改革，通过深化改革来防范和化解各种风险，通过风险管控使改革的各项举措科学依法、有力有序，加快形成适应经济发展新常态的体制机制和发展方式。四是坚持谋发展与惠民生相统一。践行以人民为中心的发展思想，坚持谋事创业都是为了让人民过上更好生活，一切为了人民，发展惠及人民。

    （一）激发需求潜力，保持经济平稳增长。坚持供给端需求端共同发力，释放需求潜力，促进经济发展。

    扩大有效投资。围绕产业转型升级、基础设施完善、新型城镇化提质、公共服务提升、创新发展提速，实施项目1000个左右，力争全年完成投资1300亿元。建立健全服务民营企业的机制和平台，深化政府和社会资本合作，引导民间资本进入城市基础建设、交通、能源、水利和社会服务领域。全力争取中央预算内投资项目和中央专项建设基金。实施项目前期分类管理，健全项目在线审批监管平台。

    积极扩大消费。深化城镇商品销售畅通、农村消费升级等 “十大扩消费行动”，发展全域旅游、健康养老、体育健身、绿色消费等新兴消费业态。实施“增品种、提品质、创品牌”专项行动，以工业消费品和食品为重点，增加高质量、高水平产品的有效供给。推进电商、快递进社区进农村，新增电商服务站点590个。

    增强出口拉动。研究制定鼓励外贸发展的政策措施，加大进出口企业出口奖励、退税、补贴力度。支持加工贸易发展。推进海关特殊监管区建设。设立河南商贸集团安阳分公司。完善跨境电商园区建设，跨境电商在外贸企业的普及率超过80%。

    服务实体经济。开展“双百”融资对接行动，引导金融机构支持实体经济。年内新增贷款160亿元，直接融资120亿元。加强50家上市后备企业培育。建立土地大储备机制，保障重点项目用地需求。支持校企合作，建立技能培训与产业发展对接机制，强化人力资源保障能力。深化“政策落实进万企”活动。建立企业首席服务员制度。弘扬企业家精神和工匠精神，以企业家素质提升引领产业素质提升。

    防控化解风险。强化企业信用监管，引导企业加强财务管控、优化债务结构、严格控制资产负债率，防控企业经营风险。成立资产管理平台公司。发挥债权人委员会作用，引导金融机构帮助困难企业渡难关。严格地方政府性债务管理，推动政府融资机制规范化、法制化。加强不良资产、企业互保等领域风险防控，开展处置非法集资决战年活动，基本完成已立案案件处置工作，守住不发生区域性系统性金融风险的底线。

    （二）提高供给质量，打好转型发展攻坚战。加大供给侧结构性改革力度，加快结构转型升级，提高供给体系质量和效率。

    抓好“去降补”。着力去产能，严格执行环保、能耗、质量、安全等法律法规和标准，加快落后产能淘汰和“僵尸企业”处置，通过改造提升一批、整合重组一批、关闭淘汰一批，推进钢铁、煤化工、铁合金、铸造、水泥、建材、有色金属等行业转型。着力去库存，优化用地结构，加快建立购租并举、市场配置与政府保障相结合的住房制度，加大货币化安置力度，鼓励农民进城购房。着力去杠杆，通过兼并重组、加快债转股、发展股权融资、盘活存量资产等方式，优化企业负债结构，降低实体企业的杠杆率。着力降成本，重点降低税负、用电、融资、社会保险、行政费用、中介评估等生产要素和制度交易成本。着力补短板，重点弥补基础设施落后、公共服务水平低、生态环境欠账多、贫困人口脱贫难等短板。

    调整产业结构。推动传统产业转型提升。组建钢铁等行业转型发展基金，支持钢铁、煤化工、装备制造、冶金建材等产业加快技术改造，拉长产业链条，调整产品结构，培育知名品牌。重点推动装配式钢结构、轨道交通、陶瓷、精细化工、染整纺织等190个工业升级项目建设。大力发展新兴产业。重点发展新能源汽车、通航制造、机器人、生物医药、节能环保、新材料等产业，支持方快锅炉、艾尔旺等企业发展壮大，推进强基精密制造产业园、新能源汽车产业园、翔宇康复设备产业园、全丰无人机、神方机器人等30个高新技术项目建设。实施光伏发电和风电“双百万工程”，新增风力发电装机容量400兆瓦、光伏发电装机容量220兆瓦。积极发展储能电站。加快发展现代服务业。重点抓好现代物流、金融保险、信息服务、文化旅游、健康养老、通用航空等产业。大力发展普惠金融，创新金融产品和服务模式，建设多层次资本市场体系。推进安西公铁物流园、欧蓓莎商贸城、红旗渠文化创意产业园等80个项目建设。

    加强发展载体建设。实施产业集聚区、服务业“两区”晋位争星行动，力争各产业集聚区在全省位次前移，新东产业集聚区达到省级门槛。推行“政府引导+市场化运作”模式，提升道路、供电、供排水、污水处理等基础设施，完善综合服务、技能培训、检验检测等公共服务平台。优化管理体制，创新工作机制，统筹运用产业、招商、财税、金融等政策，促进产业集聚，壮大主导产业。产业集聚区工业总产值突破2000亿元，服务业“两区”营业收入超过150亿元。

    推进产业融合。依托大数据云计算中心，推进数据资源开放聚集和开发利用，深入实施“互联网+”行动，扩大工业云、政务云、民生云覆盖面，大力发展网络经济。发挥铁路、公路、机场联运优势，完善枢纽功能，以物流带产业，以枢纽聚产业，大力发展枢纽经济。以节能、环保、生态产业为重点，引企业引技术，大力发展生态经济。推动“宽带安阳”“智慧安阳”建设，完善城乡信息网络基础设施。培育省、市级工业化信息化融合示范企业5家以上。

    （三）调整农业结构，做优现代农业。深入推进农业供给侧结构性改革，促进农业稳定发展、农民持续增收。

    调优种养业结构。推进“四优四化”，稳夏粮优秋种，优质专用小麦、优质油料作物、优质蔬菜、优质林果面积分别达到70万亩、50万亩、105万亩和20万亩。开展粮改饲优质草畜试点。创建市级以上标准化规模养殖场10个。新建高标准粮田30万亩。粮食总产稳定在209万吨左右。

    壮大农业产业集群。推进农产品加工专业园区建设。重点提升粮油加工、烘焙制品、高端饮品、屠宰加工等行业精深加工水平。培育林州新大牧业、汤阴万山畜牧等高成长性集群。扩大9个省级农业产业化集群规模，总收入突破300亿元。

    发展都市生态农业。深化现代农业园区创建，开发农业多元功能，抓好内黄农业科技博览园、鑫贞德光伏农牧园、太行观霖都市生态农业园等30个园区建设，新增园区面积3万亩。丰富“菜篮子”供应，创建国家级蔬菜标准园。

    深化经营体系创新。培育新型农业经营主体，推进适度规模经营。创建特色区域服务站6个。推进内黄果蔬城、龙安花木交易中心等项目建设。发展农业电商，完善鲜活农产品线上线下融合流通体系。

    强化农业生态保护。推广清洁、绿色生产模式，加强“三品一标”创建，严格农产品质量安全监管，健全农业标准化生产和农产品可追溯体系。培育优特农产品品牌。抓好大中型沼气建设。生态循环农业面积达到45万亩。实施改善农村人居环境五年行动计划，建设美丽宜居新农村。

    （四）发挥资源优势，提升文化旅游发展水平。整合文化旅游资源，理顺管理体制，形成统一、开放、高效的文化旅游业发展格局。力争全年旅游总收入突破390亿元。

    夯实旅游基础。推进龙安大道等10条旅游专线道路建设。筹建市旅游集散中心。按期完成国旅安阳城、康体文化运动园等18个项目建设任务。开通殷墟、红旗渠、太行大峡谷、岳飞庙等重点景区环线旅游车和直通车。

    培育旅游品牌。围绕殷墟、红旗渠·太行大峡谷、羑里城等品牌，打造经典游、山水游、文化游、红色游4条特色精品线路。加快殷墟国家考古遗址公园、曹操高陵保护展示工程规划建设。支持马氏庄园、二帝陵、万泉湖等景区升级改造，力争A级景区突破25家。每个县（市）区新建2个至3个乡村旅游示范点。支持林州市建设国家全域旅游示范区。推进旅游服务标准化创建，强化旅游市场监管，保障游客权益。

    加强旅游推介。启动区域旅游规划和投资运营策划工作。办好第九届航空运动文化旅游节。组织开展红旗渠畔甲骨情、中国研学旅游目的地联盟大会、国际汉字大会等活动。推动与中航集团、景域集团、凤凰古城公司、曲江集团合作项目取得实质进展。

    加快文化建设。实施文化产业“双十”工程和“文化+”工程，支持文化创意企业发展，推动文化产品创新创造。成立安阳文化影视出版集团。推进红旗渠申报世界文化遗产。促进文化事业创新发展，建成1183个基层综合文化服务中心。

    （五）加快动力转换，增强内生动力和发展活力。深入推进改革开放创新，持续添动力、增活力、拓空间。

    强化开放带动。深入对接“一带一路”、京津冀协同发展战略，深度融入省五大国家战略和新时期国家促进中部地区崛起规划。按照中原城市群省际区域性中心城市定位，突出装备制造、钢铁深加工、食品制造、文化旅游等重点领域，紧盯主导产业，分区域分行业成立专业队伍，实施产业链招商、股权招商、市场招商、并购招商。完善开放平台，抓好国家级高新区、城乡一体化示范区、产业集聚区、服务业“两区”发展，加快保税物流中心申建和铁路口岸建设。强化跟踪落实，加强与一汽、东风、北汽、陕汽、比亚迪、合众、华润等企业对接，争取合作项目取得进展。开展专项督查，确保项目履约率、资金到位率、项目开工率进入全省先进位次。在法定权限范围内制定招商引资优惠政策，优化营商环境。

    强化改革推动。打赢国企改革攻坚战。完成省属国有企业“三供一业”分离移交工作。基本完成安阳宾馆、中原宾馆以及非工业国有企业改革任务。实现市属国有企业分类监管。解决豫北棉纺厂、益康制药厂、黄河轮胎公司、织染厂改制遗留问题。加强国资监管。鼓励非公有制企业参与国企改革。深化“放、管、服”改革。继续简政放权，精简行政审批事项。全面推行权力、责任和效率清单，清理规范中介服务，开展减证便民专项行动。加快商事制度改革。实现“双随机、一公开”监管全覆盖。推动市民之家创建国家级行政审批服务标准化试点。推进“互联网+政务服务”，统一管理政府投资信息化项目，建成全市统一的网上办公系统和网上审批系统。完成市、县公共资源交易一体化，建成符合国家标准的公共资源交易服务平台、电子交易和监管平台。深化财税金融体制改革。加快综合治税信息平台建设，完善社会保险费征管新机制。推进林州市、安阳县农村金融改革试验区建设。完成林州农信社改制。深化农村改革。落实承包土地“三权分置”办法。完成农村土地承包经营权确权颁证。全面推开“两权”抵押贷款改革。继续深化水利、林业、供销改革。推进民生领域改革。以基层为重点，抓好医疗、医保、医药联动改革，推进分级诊疗制度建设，推广家庭医生签约服务，启动城市公立医院综合改革。完成城市执法体制改革。实施居住证制度。继续推进电力、价格、环保执法、文化体制、社会保障、驾驶人培训等改革。

    强化创新驱动。培育创新主体。新培育国家级高新技术企业10家，实施对外科技合作项目10个以上，引进转化国内外先进技术成果10个以上。重点支持合力创科、博奥公司等20个科技小巨人企业发展。促进创新成果转移转化。设立科技创新创业投资引导基金。创建中原“双休双创”科技示范园区。开展“百家院校科技成果直通车”和“百名专家帮百企”活动。推动中国科学院科技成果转移转化中心安阳分中心、全国技术转移公共服务平台京南基地建设。建成国家农业科技示范园区核心区。建设国家知识产权示范城市。组建科技开发投资平台、知识产权专利成果交易平台。加快安阳国家大学科技园、创新创业孵化基地建设。新增市级以上工程技术研究中心、重点实验室10个以上。汇聚创新人才。建立柔性人才引进机制。鼓励高等院校和职教院校围绕全市主导产业发展，培养实用型技能型人才。提升质量水平。启动全国质量强市示范城市创建。建立商标战略示范区（县）市、示范企业制度，争创驰名商标。

    （六）注重城乡协调，加快推进新型城镇化。坚持遵循规律、因地制宜，以人为本、公平共享，规划引领、有序推进，加快形成城乡区域协调发展新格局。

    深化文明城市创建。积极争创全国文明城市。实施“生态修复、城市修补”工程，重点抓好拆除违法建筑、整治广告牌匾、改造城市绿地、协调城市色彩、实现夜景亮化、优化城市天际线和街道立面等工作。启动新一轮城市总体规划修编。开展历史文化街区划定和历史建筑确定工作。完善部分区域管理范围和管理体制调整后代管区的规划体系。完成洹河景观规划设计、户外广告设置规划修编。强力推进违法建筑拆除。完善城市管理“十大规矩”，建立配套制度和规范标准。加快农贸市场标准化改造，有序推进市场外迁。

    完善城市基础建设。启动安阳古城保护整治复兴、文化遗址保护修缮工程。开工建设明福街、漳河大道等5个地下综合管廊。加强城市公共停车设施规划建设。推进热力管网三期配套工程。东区污水处理厂尾水人工湿地工程基本完工。开工建设垃圾焚烧发电项目。确保建筑垃圾再生利用生产线投用。推进海绵城市建设，启动消除城区重点易涝区段三年行动。

    实施棚改安居工程。启动文峰区中华路7个村、昌泰片区等22个棚改项目。棚改货币化安置比例超过50%。完成60%的新型农村社区整改任务。

    促进城区经济提速。围绕部分区域管理范围和管理体制调整，优化城区产业布局，明确主导产业定位，重点发展先进制造业、现代服务业和都市生态农业。支持殷都区发展钢铁精深加工（装配式钢结构建筑等）、精细化工、文化旅游产业，支持北关区发展纺织服装、无人机、商贸物流产业，支持龙安区发展轨道交通、灯饰灯具和生态旅游健康养老产业，支持文峰区、高新区发展研发、孵化、金融、总部经济等现代服务业，支持示范区、安阳县发展新能源汽车及零部件、通用航空、智能机器人等先进制造业和现代农业。

    推动县城建设提质。落实“百城建设提质工程”，完善基础设施，提升县城承载能力和发展水平。重点支持林州市发展电子新材料、汽车及零部件、文化旅游和建筑业，支持内黄县发展陶瓷、中医康复设备、电动车和现代农业，支持汤阴县发展食品医药产业、现代物流和特色农业，支持安阳县、示范区加快核心功能区建设。加强重点镇和特色镇建设，加快城镇基础设施和公共服务设施向农村延伸。

    构建综合交通体系。开工建设安阳机场、晋豫鲁铁路西连接线、高铁综合客运枢纽、林汝高速公路安阳段、安内快速通道。加快推进西北绕城高速公路、国道341永和至许家沟段和东北外环改建工程。完成林州通用机场、国道341楚旺至永和段、省道303安阳至姚村段改造提升工程。抓好安（阳）新（乡）郑（州）高速公路安阳段、安（阳）罗（山）高速公路安阳段、安林市域铁路等项目前期。

    （七）坚持绿色发展，加强生态环境保护治理。坚持以环保治理倒逼绿色发展，铁腕施治，铁责担当，推动治污与发展相生共赢。

    打好大气污染防治攻坚战。聚焦重点源头、重点行业、重点区域，深入推进全民治污、依法治污、科学治污、精准治污，坚决完成目标任务。一是减少燃煤污染。全面推进散煤综合治理、冬季清洁取暖，完成以电代煤、以气代煤10万户左右，农村地区清洁型煤替代全覆盖，市区建成区集中供热率达到80%。推动燃煤锅炉清洁改造，实现燃煤小锅炉“清零”。加快重点用煤企业节能改造。严格煤炭销售、煤质控制管理，加强源头防控。坚决实现煤炭消费总量负增长。二是减少工业排放。对钢铁、煤化工、玻璃、铸造、铁合金、水泥、有色金属等7个重点高排放行业，实施“一企一策”深度治理、升级改造。严格执行特别排放限值和排污许可管理。开展化工、工业涂装、包装印刷等行业挥发性有机物综合治理。所有涉气重点企业实行驻厂监察、24小时在线监控。科学规划专业园区，有序推进市区建成区重点涉气工业企业搬迁。全面取缔“小散乱污”企业。三是减少扬尘污染。所有施工工地实施监督员、网格员、管理员“三员”管理，严格落实“八个百分百”。提升城市道路清扫、保洁机械化精细化水平，严格“以克论净”考核。四是减少车辆排放。全部淘汰黄标车。实施大货车、重型柴油车常态化管控。推广使用新能源汽车，推进公交车、出租车“油改气”。全面供应国六标准燃油。五是有效应对重污染天气。加强分析研判预警，全面核定各类污染源排量，科学精准制定应急措施，严格落实不同响应级别下清单式管控。完善网格化“一长三员”管理，做到网格全覆盖、巡查全天候，管住管好每个污染源。六是严格环境执法和监督问责。切实强化政府监管责任和企业主体责任。严格查禁露天焚烧、违规燃放烟花爆竹行为。全面清除黑加油站点。对偷排、造假的，必须严厉打击；对执法不力、姑息纵容的，必须严肃追究；对空气质量恶化、应对不力的，必须严格问责。治理雾霾人人有责，贵在行动、成在坚持。全社会不懈努力，蓝天必定会一年比一年多起来。

    加强水、土壤污染防治。推进工业废水达标排放。加快污水处理设施建设，完善污水收集管网，实施城区2条河流、10条沟渠、8个坑塘治理，完成570个入河排污口截流工作，基本消除市区建成区黑臭水体。在市区建成区、县城、中心镇和重点流域设置畜禽、水产养殖禁养区。抓好集中式饮用水源地环境保护。减少地下水采用。全面落实“河长制”，一河一策，系统推进水污染防治、水生态保护和水资源管理，确保河流水质优良比例总体达到37%。防治农业面源污染。编制土壤污染防治工作方案，加强土壤污染治理与修复，严格危险废物环境监管。支持农村集中连片综合治理、河道生态修复和垃圾集中处置。推进大宗固废综合利用和城市矿产回收再利用，支持红旗渠经济技术开发区循环化改造。

    加强生态保护和建设。推进林业生态市提升工程，启动国家森林城市创建。营造林16.4万亩，实施太行山绿化9万亩、平原林网提升20万亩。加快生态廊道建设，提升京港澳高速公路城区段、南水北调生态带绿化水平。推进3个国家湿地公园试点建设。加快水系建设。开工建设洹河于曹沟下段和辛村段、汤河周流段、粉红江磊口段等河道综合治理工程。实施红旗渠、跃进渠、漳南、大功等灌区节水技改工程。完成南水北调中线防洪影响处理工程。确保引岳入安二期供水主管道和东湖闸、西湖闸主体建成。

    （八）切实改善民生，维护社会和谐稳定。加大民生投入，办好民生实事，不断增强人民群众获得感。

    强力推动脱贫攻坚。严格落实脱贫攻坚责任制，持续打好“转、扶、搬、保、救”组合拳。扩大光伏、旅游、特色种养等产业扶贫覆盖面。实施贫困村整体提升工程，推动教育、交通、医疗、水利、电力等5个专项方案落实，确保64个贫困村实现“七通七能七有”。统筹做好非贫困村贫困人口脱贫工作。推动“六改一增送温暖”活动常态化。确保21400人稳定脱贫、1333人易地搬迁脱贫。

    稳定扩大社会就业。突出做好高校毕业生、农村劳动力、去产能企业分流职工三大群体就业工作，做好军队转业干部、退役士兵接收安置工作。促进“零就业家庭”成员实现就业。推动创业带动就业，重点支持大学生和返乡农民工创业。新增农村劳动力转移就业3万人。

    完善社会保障体系。实施全民参保登记计划。深化机关事业单位养老制度改革。调整退休人员基本养老金、城乡居民基本养老保险基础养老金最低标准。完善失业保险制度。落实企业年金和职业年金管理办法。全面实施统一的城乡居民医保制度，提高基本医疗保险财政补助标准。完善城乡居民大病保险制度，落实困难群众大病补充保险制度。城乡低保月人均补助水平分别提高到250元和142元，孤儿集中供养、分散供养标准分别提高到1100元和700元。健全社会救助、社会福利和养老服务体系。完成市儿童福利院搬迁。加强农村“三留守”人员关爱服务和困境儿童保障，切实兜住民生底线。

    大力发展社会事业。促进义务教育均衡发展。扎实推进165个“全面改薄”项目，扩充城镇义务教育资源，优化中小学布局，新建改扩建中小学18所、幼儿园69所，努力让学校建设与城市发展相协调。加快发展现代职业教育。支持规范民办教育发展。深化健康安阳促进行动。完善医疗卫生服务体系，加强与京津等地区知名医院合作，建设区域医疗中心，确保县级疾控中心、妇幼保健院和乡镇卫生院等10个项目建成。深入推进文化惠民工程。广泛开展全民健身，提高竞技体育水平。继续实施老年体育长青工程。

    加强社会治理创新。全面落实安全生产责任制，保持“打非治违”高压态势，确保不发生重大安全事故。加强食用农产品、进口食品和药品监管，建成食品安全监测中心。完成第九届村委换届。建成标准化社区70个。积极构建综合防灾减灾救灾体系，提升灾害应对能力，全面完成“7·19”灾后重建任务。完善信访工作机制，加强矛盾纠纷调处化解，做好重大项目社会稳定风险评估，及时预防化解各类群体性事件。强化社会治安综合治理，建设智慧视频云监控系统，加快构建立体化社会治安防控体系，完善反恐和打防犯罪机制，确保社会和谐稳定、人民安居乐业！

    加强社会主义核心价值观建设，大力弘扬焦裕禄精神和红旗渠精神，学习宣传时代楷模许帅先进事迹，深化行业道德实践，开展文明社区、文明家庭等群众性精神文明创建活动，提升文明素质，打造文明安阳。支持国防和军队建设改革，加强民兵预备役建设，支持人民防空建设，推动军民融合深度发展。加强老区建设。支持工会、共青团、妇联等人民团体发挥桥梁纽带作用。认真做好老龄和关心下一代工作。继续做好统计、史志、档案、气象、地震、邮政、发展研究、外事侨务、民族宗教等工作。

    办好事关人民群众切身利益的“十件实事”：

    1.实施118条市政道路建设。其中，建成永明路北段、迎春东街、钢三路等50条道路，完成铁西路南段、商都路等15条道路大修改造。

    2.规划实施迎宾公园、洪河公园、龙安公园等190个园林绿化项目，开工建设165个，年内完成153个。

    3.开工建设市一中新校区、市实验幼儿园新园。确保市六十九中竣工。招聘农村特岗教师300名。

    4.开工建设第五人民医院病房楼、第二人民医院新院、残疾人康复中心。

    5.开工建设中国文字博物馆续建工程、市文化艺术中心、市体育中心。

    6.新增新型公交车50台。新增城市公共自行车3000辆。

    7.开工建设第六水厂。

    8.解决所有贫困村安全饮水问题。

    9.实施县乡公路三年提升计划，今年新改建农村公路300公里。完成中心村电网改造升级、农田机井通电和贫困村通动力电工程。

    10.新开工棚户区改造13780套，基本建成棚户区改造和公共租赁住房16000套。

    三、加强政府自身建设

    加快发展，振兴安阳，人民寄予厚望，政府责任重大。必须坚持不懈加强政府自身建设，提升政府行动力、执行力和感召力。

    （一）依法行政，重法治。全面贯彻法治政府建设纲要，把依法行政、依法办事贯穿于政府工作全过程。全面履行政府职能，法定职责必须为，法无授权不可为，做到管理、服务、执法都在法治框架内有序运行。提高立法质量，严格行政执法，加强执法监督。深入推进“七五”普法。全面建立政府法律顾问制度。自觉运用法治思维和法治方式深化改革、推动转型、破解矛盾、维护稳定。全面深化政务公开。自觉接受人大工作监督和法律监督，接受政协民主监督，接受社会监督和舆论监督，确保政府运行公开阳光透明！

    （二）解放思想，破难题。始终保持思想的敏锐性和开放度，切实把创新、协调、绿色、开放、共享发展理念贯彻到政策制定、工作安排、任务落实各领域各环节。以新发展理念检视本地区、本部门、本单位的工作和发展实践，坚决摒弃和破除不适应不符合甚至违背新发展理念的思想认识和行为做法。对经济社会发展体制机制障碍、人民群众关心的热点难点问题、“四大攻坚战”重点任务，找准症结，专题化解，专案突破，坚决打掉工作落实中的“拦路虎”！

    （三）转变作风，提效能。牢记为民宗旨，夙夜在公，毫不懈怠，一门心思抓项目、促落实，真心真力抓发展、惠民生。严格执行工作责任制，对确定的目标、敲定的项目、议定的事项，一一建台账、细分解、明责任、定时限、严督导，“挂图作战”，拼在一线，以上率下，一抓到底。加强公务员队伍教育培训。推动政府系统学先进、比创新、看实绩。完善考核机制，强化奖优罚劣。加强督查问责，严厉整肃庸政懒政怠政行为。全面落实容错纠错机制，给干事者鼓劲，为担当者撑腰！

    （四）廉洁从政，守规矩。始终在思想上政治上行动上同以习近平同志为核心的党中央保持高度一致，切实增强“四个意识”，思想上深刻认同核心，政治上坚决维护核心，组织上自觉服从核心，行动上坚定紧跟核心。严守政治纪律，恪守政治规矩，保持政治定力。认真落实全面从严治党要求，坚决贯彻中央八项规定精神，严格执行《准则》和《条例》，一以贯之刹“四风”、正作风。完善财政资金使用、国有资产监管、公共资源交易等制度。加强行政监察、审计监督，坚决惩治侵害群众利益的不正之风和腐败问题，以反腐倡廉实效取信于民！

    各位代表！

    行动改变安阳，实干铸就辉煌。让我们更加紧密地团结在以习近平同志为核心的党中央周围，在市委的坚强领导下，不忘初心，继续前进，埋头苦干，开拓创新，确保完成经济社会发展各项目标任务，用实际行动谱写重返全省第一方阵、决胜全面小康、建设区域性中心强市新篇章，以优异成绩迎接党的十九大胜利召开！
